# Synaptic Scaling Enables Dynamically Distinct Short- and Long-Term Memory Formation

**DOI:** 10.1371/journal.pcbi.1003307

**Published:** 2013-10-31

**Authors:** Christian Tetzlaff, Christoph Kolodziejski, Marc Timme, Misha Tsodyks, Florentin Wörgötter

**Affiliations:** 1 Faculty of Physics – Biophysics, Georg August University Friedrich-Hund Platz 1, Göttingen, Germany; 2 Network Dynamics Group, Max Planck Institute for Dynamics and Self-Organization, Göttingen, Germany; 3 Bernstein Center for Computational Neuroscience, Georg-August-University Friedrich-Hund Platz 1, Göttingen, Germany; 4 Faculty of Physics – Nonlinear Dynamics, Georg August University Friedrich-Hund Platz 1, Göttingen, Germany; 5 Department of Neurobiology, Weizmann Institute of Science, Rehovot, Israel; Duke University, United States of America

## Abstract

Memory storage in the brain relies on mechanisms acting on time scales from minutes, for long-term synaptic potentiation, to days, for memory consolidation. During such processes, neural circuits distinguish synapses relevant for forming a long-term storage, which are consolidated, from synapses of short-term storage, which fade. How time scale integration and synaptic differentiation is simultaneously achieved remains unclear. Here we show that synaptic scaling – a slow process usually associated with the maintenance of activity homeostasis – combined with synaptic plasticity may simultaneously achieve both, thereby providing a natural separation of short- from long-term storage. The interaction between plasticity and scaling provides also an explanation for an established paradox where memory consolidation critically depends on the exact order of learning and recall. These results indicate that scaling may be fundamental for stabilizing memories, providing a dynamic link between early and late memory formation processes.

## Author Summary

The ability to form memories of the past is a main feature of the brain. Memories are formed by learning. However, the biological mechanisms for learning, which change the synaptic weights by synaptic plasticity, act on a different time scale (minutes) than those that lead to memory consolidation (days). Experimental results of the last 15 years show that there exists another mechanism, named synaptic scaling, which also influences synaptic weights but on an intermediate time scale (hours). In this study, we analyse whether this process could bridge the time gap and to what degree it can be used to link the processes of synaptic changes with the slow processes of memory formation (and forgetting). Furthermore, the combination of synaptic plasticity and scaling provides a possible explanation for the effect that memory recall can destabilize existing memories. Thus, our results suggest that synaptic scaling is a fundamental mechanism for the dynamic processes of memory.

## Introduction

Memory function consists of different, temporally overlapping stages, roughly divided into working memory, short-term and long-term memory, which are distinguishable by their increasing capacity and storage duration [1], [2]. Especially long-term memory requires lasting changes which involve synaptic plasticity and, subsequently, other complex and slow physiological and anatomical network processes. Furthermore, the formation of long-term memories relies on memory consolidation ([3], for a review see [4]). Consolidation, in turn, seems to rely on the intrinsic activation of the network that happens during sleep [5]–[7]. Commonly one distinguishes between two types of consolidation [4], [8]–[10]: (i) systems consolidation which transfers memories from one brain area to another (e.g., from hippocampus to neocortex) and (ii) synaptic consolidation which stabilizes memories within a brain area. However, even after consolidation, memories are not ‘frozen’, thus, new memories learnt can disrupt memories previously learnt and, furthermore, the recall of a memory can destabilize this memory [4], [11]–[13]. Memories have to be (re)consolidated several times to achieve permanence [4].

It is an intriguing problem how the nervous system is capable of distinguishing between memories of different storage duration within the same brain area. Given that memories are represented by synapses [14], [15], somehow candidate synapses for long storage duration (named in the following long-term storage LTS to not confuse this with long-term memory) must respond differently to those that are involved in short-term storage (STS) only. In particular, one would expect that LTS-candidate synapses should be susceptible to synaptic consolidation, while STS-candidates should not.

All this happens mainly in the cross-section of the hippocampal and cortical networks, a highly dynamic system continuously driven by inputs as well as by intrinsic activity patterns. In spite of this dynamic volatility, the network is capable of maintaining the synaptic integrity of LTS-candidates for a long enough time such that systems consolidation and other processes can set in.

Many computational and psychological memory models describe the dynamics of systems consolidation between hippocampus and neocortex by introducing different time scales for plasticity [16]–[20]. By contrast, experimental evidence [21] indicates that the time scales are about the same. For synaptic consolidation the underlying central difficulty, which makes it hard to design more realistic memory models, is that synaptic plasticity operates at time-scales of seconds to minutes while consolidation takes days. The first steps after memory formation are the processes of protein synthesis [4] and tagging [22]–[24] distinguishing short- from long-term plasticity. They occur on a time scale of minutes to hours after learning. However, synaptic consolidation consists of several steps [4], [10] and experimental evidences point out that NMDA- and AMPA-receptor reactivations [25]–[27] and sleep [6], [28] are needed even days later to (synaptically) consolidate a new learnt memory. Thus, there is a time-gap between neuronal physiology (synaptic plasticity; minutes) and consolidation (days). A physiologically plausible, fully dynamic memory model that bridges such time-spans (from learning to consolidation) such that LTS-candidate synapses properly respond to synaptic consolidation, while STS-candidates do not, is still missing.

Here we work towards bridging this gap by considering one additional, well-established physiological component which naturally operates at a longer time scale: synaptic scaling [29]. Synaptic scaling has primarily been associated with the homeostatic regulation of activity in a network [30]. Overly active networks will – on a time scale of hours up to days – down-scale their activity and vice versa, which is a result of synaptic scaling, where synaptic weights are regulated by the deviation from a homeostatic level of activity.

In the following, we show that neural circuits, which combine synaptic scaling with conventional plasticity [31], [32] such as long-term potentiation (LTP; [33]), long-term depression (LTD; [34]), or spike-timing-dependent plasticity (STDP; [35]), naturally exhibit a transition from short- to long-term storage, where LTS-candidate synapses are consolidated and maintain their integrity through unspecific, “sleep-like” activation, while STS-candidates fade. This bi-modal characteristic is due to an intrinsically arising nonlinearity that induces – without any addition assumption – a natural bifurcation in the dynamics of the system. Intriguingly, this bifurcation can also explain experimental results [36] on the apparently paradoxical effect of memory destabilization during reconsolidation protocols [11], [37], where the recall of a previously learnt aspect actually disrupts its memory. Our model does not attempt to implement any of the complex and still little understood mechanisms for systems consolidation or other long-term processes, which would lead to true long-term memories. Instead, the goal of this study is to present a generic mechanism for dynamically maintaining synaptic integrity of LTS-candidates in the network by synaptic (re)consolidation. Thus, this study suggests a solution to the long standing problem of synaptic stability in a fully dynamic network by proposing a bifurcation scenario resulting from combined plasticity and scaling.

## Results

Substantial evidence exists that strong synapses can maintain their integrity better than weak ones, which are, for example, more easily pruned during developmental processes [38], [39]. Here we show that this might not just be due to the quantitative difference in synaptic strength. Instead such synapses may follow qualitatively different dynamics in networks with long-term plasticity and synaptic scaling.

### Two different time scales of memory

Consider, for instance, a model neural circuit (see Materials and Methods) of locally connected rate-coded units. Each unit 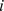 is described by a leaky membrane potential 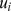 and a firing rate or activity 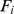 which depends in a non-linear way (here sigmoidal) on the unit's actual membrane potential (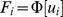). This formulation allows for a general interpretation of each unit as either a rate-coded neuron [40], [41] or a population of neurons [42]–[44]. Thus, the here presented results are independent of the spatial scale of the neural circuit. In the following, we will use the terms ‘unit’ and ‘neuron’ synonymously.

**Figure 1:**
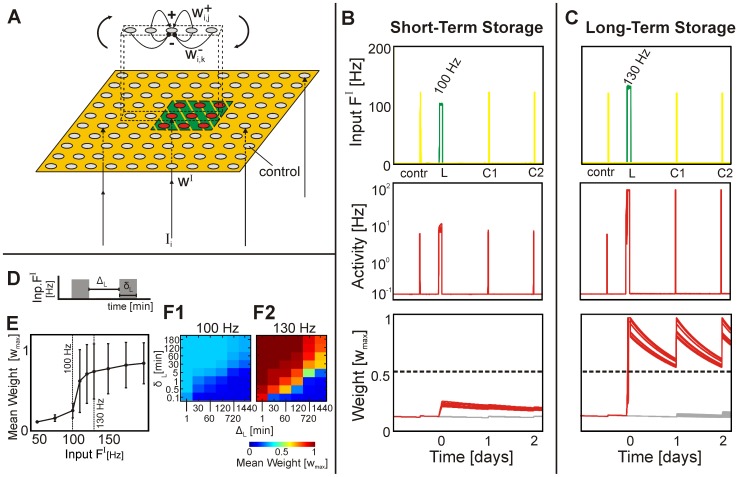
Increasing the input frequency yields synapses that recover their weight by global, consolidation-like stimulation. (**A**) The network consists of a square grid of 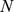 units with periodic boundary conditions in both directions. Each unit connects excitatorily with its nearest neighbours (see purple area regarding blue neuron) and inhibitorily with the nearest and next-nearest neighbours (purple and bluish gray area). Each unit receives an external projection (only a subset is shown). Two different input types are delivered: (i) a local learning stimulus (‘L’, green area) and (ii) a global input to all neurons (‘C’, yellow). (**B,C**) Different input intensities induce different activities (middle row) and weights (bottom row) of the input-target neurons (red). Pulses for local learning L are 5–10 times longer than for global consolidation C stimuli (see panels D–F for accurate stimulation-response details). Before learning short activation of all neurons (‘contr’) has no significant effect on the weights. (**B**) Learning signal L with 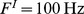. Synaptic weights of the red neurons grow but not the control weights (gray). After learning all activities relax back to background (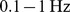) and weights decay. Subsequent consolidation stimuli (C1,C2; 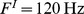) change weights minimally. (**C**) Stronger learning signal L (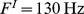) induces stronger weight growth (red curve) than in B. Now consolidation pulses (C1,C2; as before) yield weight recovery. This happens for all stimuli that drive weights across the bifurcation level of weight decay versus recovery (dashed horizontal line). (**D**) Stimulation protocol during learning. (**E**) Mean synaptic weight shows for increasing inputs an abrupt transition (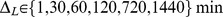 and 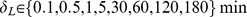). (**F1,F2**) Different combinations of input interval 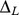 and duration 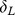 robustly lead to the same weights (red neurons) for different input intensities (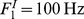, 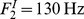). **B–F**: Background input has an intensity of 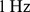 and all inputs are noisy (see Materials and Methods).

In the basic state every neuron receives a small noisy background input of about 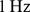. For a certain period of time (here about two hours), only a local patch of neurons receives an external input of stronger intensity (see green striped area in Figure 1 A and green pulse ‘L = Learning’ in Figure 1 B,C; all inputs are noisy) while others do not and serve as control. This input mimics localized rate-coded signals from the environment or other brain areas delivered to the circuit. In the circuit plastic excitatory synapses to the nearest neighbor neurons exist (see, for instance, in Figure 1 A the purple area regarding the blue unit), as well as, short- and long-range lateral inhibition with unchanging synaptic strengths (purple and bluish gray area). For simplicity, we assume that each unit provides excitatory and inhibitory synapses. The dynamics of the excitatory synapses 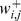 between neuron 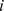 and 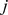 is governed by the combination of synaptic plasticity and scaling defined as [31]:
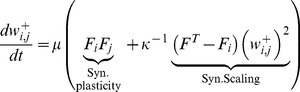
(1)where 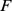 is the activity, 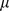 a time constant of synaptic changes, 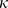 the ratio of plasticity and scaling time constants, and 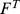 the desired homeostatic level of activity. As shown in previous work [31], [32], the quadratic weight-dependency of the synaptic scaling term guarantees convergent synaptic weights without the need of additional constraints [45]–[49]. The synaptic plasticity part consists only of a correlation-based LTP-term. Analytical and numerical results demonstrate (see below and Text S1) that a synaptic plasticity rule consisting of a combination of LTP and LTD does not alter the general dynamics, we will discuss in the following.

Depending on the intensity of the external input, differently strong synaptic weights between the stimulated units are induced by the combined rule of plasticity and scaling (bottom panels in Figure 1 B,C). Thus, the units of the stimulated patch form a local cell assembly similar to those found in recent experiments [50]–[52] and represent a memorized version of the local external input. Small differences in input intensity (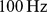 vs. 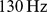) induce large differences in weights (bottom panels, red curves). The gray curves represent the controls from neurons that do not receive the strong external input. As we show below, these strong weights differences (red curves) arise from a generic nonlinear property of the network, where weight-formation follows a saddle-node bifurcation. This nonlinearity exhibits an intriguing phenomenon: When all units in the circuit (within and outside the cell assembly) receive a strong (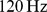) but brief input (here about 15 minutes; yellow needles, ‘C1,C2 = consolidation’, in panels B,C) only the strong synapses will recover (panels C), while the weak ones continue to decay (panels B). Here this brief and global input takes the role of the coherent, but unspecific neural activation during slow-wave-sleep, which is commonly considered as a potential basis of synaptic consolidation [5], [7]. This observation is the first indication that the combination of plasticity and scaling in a simple dynamic model allows differentiating between synapses for short-term storage, which decay, from those for long-term storage, which can be recovered (or rather consolidated).

Furthermore, we note that the network has only increased activity during external stimulation. Such a stimulation yields an imbalance in neuronal circuit activity depending on the recurrent synaptic weights. Thus, the learnt cell assemblies are stronger activated than controls and the memory contents stored in the network are read-out (see below). As soon as the external input is not present any more and only background input remains, all activities relax back to background firing rate (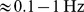) although recurrent weights are still high (Figure 1 B,C). This is an important difference to attractor memory models [53]–[55], which will continue to be active after stimulus withdrawal for (theoretically) infinitely long time. This persistent activity is important for explaining the dynamics of working memory (seconds) but contradicts the idea of long-term memories which are not permanently active. Here, the memory content is transferred from the input to the synaptic weights [14]. The activities can relax back to background state.

We remark that the emergence of the here shown phenomena does not rely on saturation effects and fine tuned topology (see Text S1). A detailed quantification is provided below. First, we show the impact of a memory recall on the spatial structure of the LTS-synapses.

### Learning and recall

**Figure 2:**
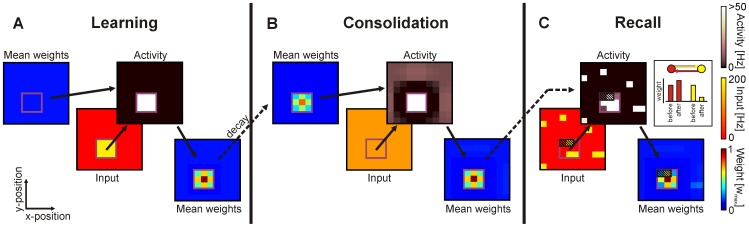
Spatial structure of activity and weights during learning, consolidation and recall. (**A**) A local learning input (region marked by purple squares) leads to growth of all input driven weights. Mean weights are plotted, which naturally are smaller for border or corner neurons as they do not get inputs from outside. (**B**) Before consolidation, weights have decayed but will be recovered fully by a global and weak consolidation stimulus given to the whole network. (**C**) Recall stimulates only some of the input neurons. Nonetheless, activity is filled in and the memory pattern is completed. Note, an imbalanced activation induces a disparate development of weights between strongly and weakly active connected neurons, for example those marked by hatching in panel C. One weight shrinks substantially (see inset in C, yellow bars).

During recall the spatial distribution of weights and activities reveals an interesting competitive effect (Figure 2), that is important for the formation of *different* memory cell assemblies and also leads to the paradox of memory loss during recall ([36], see below). Initially, during learning only the a local patch of units is stimulated and the synapses of their target neurons all grow (purple square in Figure 2 A; L-phase in Figure 1 C), where we have used a strong and local stimulus to drive all synapses into the LTS-regime. Consolidation stimulates the complete network and all synapses within the assembly recover or exceed their initial strengths (Figure 2 B; C1,C2-phase in Figure 1 C). The process of remembering (recalling) a memory is often understood as partial stimulation of an assembly and potentially of some other neurons [56]–[58]. By ways of its learnt connections the assembly produces a filling-in and generates a spatially quite complete excitation pattern including most of its members (so-called pattern completion). According to the literature [1], [14], [56]–[60] this represent the behaviorally relevant recall activity. Therefore, only a randomly selected subset of assembly-neurons receives a stimulation (we used here about 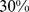 with some outliers). The resulting network activity clearly shows a filled-in spatial assembly structure (Figure 2 C; Please note that due to the partial stimulus all units of the assembly are stronger active than the control ones. Thus we can assume that the assembly is completed.), where, however, sometimes strongly active neurons are neighbors of weakly active ones. For such constellations the different activities induce a dissimilar weight dynamic. Consider a pair of mutually connected neurons (see hatching in panel C). The weakly active neuron (but still more active than controls) induces a small synaptic plasticity term and synaptic scaling is weak, too. By contrast, the synaptic scaling term for the strongly active neuron is large and, thus, dominates the dynamics. As a consequence, the corresponding weight shrinks substantially (Figure 2 C, inset, yellow bars; see also Text S1 for equations).

We remark that such network structures with generic lateral inhibition admit separation of different assemblies from each other if learning stimuli do not overlap too much. On the other hand - as soon as overlap exists - activation imbalances, as described above, may lead to interference and competition between different memories. The consequence of this will be discussed in conjunction with the paradox of memory loss during recall [36] at the end of this study.

### Analyzing STS- and LTS-domains

The difference between STS- and LTS-synapses in Figure 1 is a non-linear phenomenon, which is due to a saddle-node bifurcation and as such robust against changes in the stimulation patterns, representing different learning protocols. We tested a range of different input strengths and pulse protocols (Figure 1 D). Generally, for small external inputs the resulting synaptic weights depend roughly linear on the intensity (Figure 1 E) with a sudden jump to high values above a certain input intensity. The critical value, where this transition takes place, is insensitive to details in the pulse protocol (indicated by the strong weight differences shown in Figure 1 F1,F2).

The mechanism inducing this phenomenon is readily understood by investigating the dynamics of this system in more detail. We first analytically calculated the characteristic Weight-Input curve of this system.

**Figure 3:**
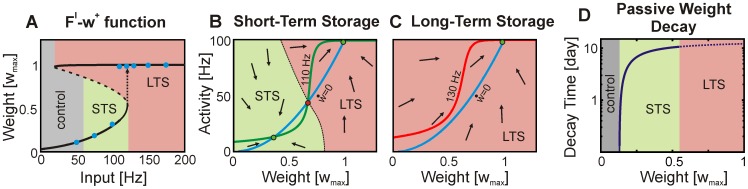
Combination of plasticity with synaptic scaling generates two distinct weight domains representing short-term and long-term storage. (**A**) Both domains (STS and LTS) arise from a bifurcation (see main text and Text S1) between two stable fixed point domains (continuous lines). Depending on the input, weights either continuously grow (control and STS-domain) or suddenly jump to a high value (LTS-domain). Between both domains there is a transition range (dashed line). Blue dots show results from numerical simulations (Figure 1), which match the analytical curve. (**B**) Fixed points are defined by the intersection between activity- (green) and weight- (blue) nullclines. As long as all three fixed points (green stable; red unstable) exists the phase space is divided into two attractor regimes which are also indicated by arrows (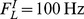). (**C**) Higher frequencies shift the activity-nullcline (red line) upwards which results in only one attractor regime (LTS; 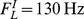). (**D**) Passive weight decay happens for all synapses as long as there is no consolidation stimulus present. Dashed parts of the curve indicate that LTS-synapses can be consolidated.

In the following we will show in an abbreviated form the analytical calculations (see Text S1 for more details). We assume that the long-range inhibition separates the circuit into two (or more) subnetworks: (i) the externally stimulated local patch(es) and (ii) the unaffected control units. This enables us to average Equation 1 over all units within such a subnetwork. To calculate the fixed point of the resulting mean field differential equation we set it equal to zero and solve it. As result we receive the weight-nullcline of the system (The weight-nullcline is a set of states where weights do not change under the given dynamics.):
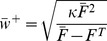
(2)with 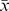 as averaged value of variable 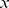. Equation 2 describes the resulting strength of the synaptic weights within a subnetwork given the dynamic of plasticity and scaling and a mean neuronal activation 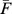. As the maximal activation of each unit can not exceed 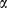 (given by the input-output function 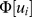), the maximal possible synaptic weight is given by 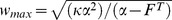. The resulting weight-activity function in the phase space is shown in Figure 3 B,C (blue line) for the parameters used in Figure 1. Of course, the course of the function depends on the used synaptic plasticity rule (the numerator in Eq. 2), but it also shows that the LTP-term (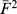) dominates and that additional plasticity mechanisms (e.g., LTD [34] or short-term plasticity [61]) do not alter the basic dynamic (see Figure S1 in Text S1).

The average activity within a subnetwork induces certain synaptic strengths (Equation 2). In turn, the mean external input 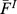 (multiplied by the input weight 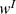) and the average recurrent synaptic weights themselves adapt the average activity. The resulting fixed point of this dynamic is calculated by the mean field differential equation of the membrane potential 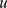 (Eq. 4). This yields the activity-nullcline (In analogy to the weight-nullcline, the activity-nullcline is a set of states where activities do not change.):
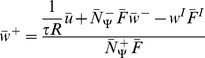
(3)with membrane resistance 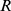 and average excitatory (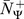) and inhibitory (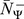) number of connections per unit within the subnetwork. As 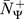 and 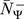 are the only topology-related parameters in this equation (and Eq. 2), the described dynamics are independent of the detailed topology (see Figure S2 in Text S1). The activity-nullcline follows roughly the sigmoidal shape of the activation function (Eq. 5). Furthermore, it shows that external inputs of different intensity delivered to the circuit change the neuronal activation (see green line in Figure 3 B for 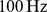 compared to the red line in panel C for 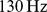) and, therefore, (via Eq. 2) the synaptic weights. The direct influence of the external input on the synaptic weights within a subnetwork can be assessed by calculating the intersections between both nullclines. These intersections are the fixed points of the whole subnetwork (activity as well as weights). The resulting fixed point equation has no closed-form solution and, therefore, has to be solved numerically. Direct simulations of the whole circuit (Euler-method) match our theoretical predictions (Figure 3 A).

**Figure 4:**
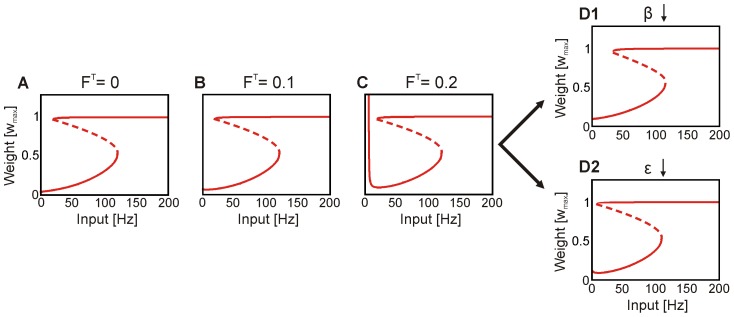
Robustness of bifurcation structure. (**A**) 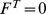: The 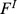 vs. 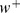 function of the fixed points of the system as already shown in Figure 3 A. For simplicity we show here only the curve without indicating the different storage domains. (**B**) 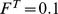: Changing, for instance, the desired firing rate parameter of the synaptic scaling term does not induce significant changes in the 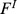 vs. 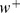 function. The overall circuit dynamics are the same as shown in Figure 1 (see Figure S3 in Text S1). This holds for negative 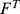 values (not shown), too. (**C**) 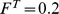: Only a dramatically different 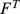 value induces changes in system's dynamic. Here, a pole emerges for small input intensities. To avoid this pole and maintain the desired dynamic the background input could be increased (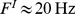) to keep the system on the right side of the pole. Alternatively, other parameters could be adapted. For instance, (**D1**) the steepness of the neuronal output function (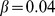) or (**D2**) the inflexion point (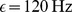) have to be decreased.

Specifically, we find a saddle node bifurcation where different fixed points are reached for low as compared to high input intensities. For the particular setting displayed in Figure 3, a continuous regime of fixed points for the weights exists for firing rates below approximately 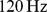 (Short-Term Storage, STS; green, Figure 3 A), while above this frequency, the system jumps to a fixed point regime with substantially larger weights (Long-Term Storage, LTS; red, Figure 3 A). The gray area below STS represents the range of weights found for the randomly stimulated control neurons (targets of the yellow neurons in Figure 1 A). Note, to obtain this curve we assumed that the circuit consists of several roughly independent subnetworks. This means that in one circuit different fixed points are reached at different spatial locations. For instance, in Figure 1 C after local stimulation the (local) patch is in the LTS-regime (about 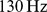 in Figure 3 A) while the control units are weakly stimulated and, therefore, they are in the gray control regime (about 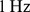) with small synaptic weights. The bifurcation is essential for the dynamics discussed here. Using different parameter values for the system does not change the fixed point curve significantly (see, e.g., Figure 4 B and Figure S3 in Text S1 compared to the used setting shown in Figure 4 A and Figure 1 B,C). However, if one parameter is changed dramatically an adequate adaption of the other parameters can still guarantee the desired circuit dynamics (see Figure 4 C,D). Thereby, the range of parameters remains in a physiological regime.

The emergence of the (desired) form of the 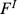 vs. 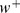 function can be explained by the changing locations of the nullclines in the phase space (Figure 3 B,C). For small input frequencies, the nullclines intersect at three different points and, therefore, two stable and one unstable fixed points exist (green and red markers in Figure 3 B). As weights gradually start to grow from low values, the system gets trapped in the lower stable fixed point in the STS-domain. For high input frequencies only one stable fixed point exists which is in the LTS-domain (Figure 3 C). As soon as the strong external input ends, only the lower fixed point exists and the weights start to decay and, without further inputs, reach control values after maximally ten days (Figure 3 D and Text S1). However, brief consolidation inputs prevent this as discussed next.

### Consolidation of memory

**Figure 5:**
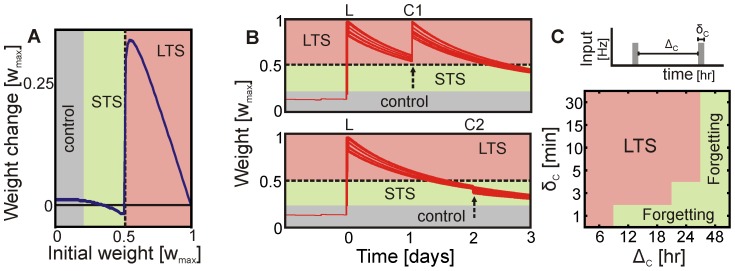
Consolidation qualitatively relies on synaptic strength and temporal protocol. (**A**) The global activation signal (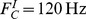, 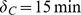) induces the recovery of those synaptic weights which are above the bifurcation threshold (LTS-regime; dashed line). For synapses just under the threshold consolidation mildly decreases the weights. Decrease arises from the network inhibition (network as in Figure 1 A). (**B**) Recovery of weights occurs only if the consolidation stimulus occurs early enough (C1; upper panel). When the stimulus is too late (C2; lower panel), weights have dropped into the STS-regime and cannot be recovered. (**C**) Weight recovery is robust to changes of interval 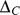 and duration 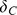 of the consolidation signal (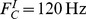, 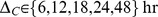 and 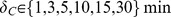). For intervals up to 24 h relatively short consolidation signals – either delivered as one or as many pulses – suffice for recovery.

Bifurcation analysis also helps to understand why synapses with values in the upper fixed point regime (LTS-synapses) respond to global and unspecific consolidation inputs while others do not. Weight changes strongly differ for differently strong initial weights when presenting a single consolidation stimulus (Figure 5 A and Figure S6 in Text S1). Weights above the bifurcation threshold (dashed line) are increased substantially, while those under the threshold are almost unaffected (close beneath threshold they rather decrease due to the lateral inhibition, see Figure 1 A, top). This phenomenon is robust against the duration of the consolidation stimulus (Figure S4 in Text S1). As a consequence, while all weights decay after learning, consolidation will recover those above bifurcation threshold. Hence, consolidation must not come too late, or also those weights might have dropped beneath threshold from which they cannot be recovered (Figure 5 B). Note, this phenomenon is not “history dependent”, which means it does not matter whether learning or consolidation had driven the weights into the LTS-regime before decay has set in (Figure S5 in Text S1).

We remark that our model solely captures dynamic network effects and that we do not attempt to model systems consolidation, which relies on complex and little-understood physiological processes. It appears, however, important that the here observed dynamic properties of such a network allow synapses to maintain (and regain) their stability such that systems consolidation or other processes may find a stable substrate to operate on. The wide parameter range within which this happens (Figure 5 C) supports this argument, because recovery is robust and stable. Only if the consolidation input is too short or too late, forgetting sets in.

As consolidation is a sleep-induced effect [5], [6], [10], little is known about the actual activity characteristics of the consolidation process. Input intensities required for consolidation are similar to those for initiation (similar to Figure 1 E), but emphasis lies on the fact that for consolidation the whole network is stimulated in an unspecific way and that the consolidation stimuli can be shorter (in Figure 1 B,C about 15 minutes of total duration). Additionally, similar to during sleep induced activations (e.g., spindles or ripples [6]), the memory-related cell assembly is reconstructed (“replayed”; see, e.g., [62] for review) during the consolidation input (Figure 2 B).

### Stimulus-dependent destabilization of memory

The recall of a previously well-learnt memory item may lead to the paradoxical phenomenon that this memory will be less well remembered than a newly learnt one. In the literature, this phenomenon is widely interpreted as memory destabilization or rather disruption [4], [11]–[13], [37], [63] and has been found in some studies [11], [64], but not in others [65], [66]. Thus, the question arises what the dynamical processes are that underlie it and especially also why memory destabilization/disruption depends on details of experimental protocols. In one specific experimental paradigm [36] destabilizing happens due to the interference of a new memory item with the previously learnt first memory, but only if the first memory was recalled before the second was learnt. In this protocol the first memory is impaired, while the new one is now susceptible to consolidation. In the following, we show that combined plasticity and scaling also naturally accounts for this paradox. We compare the experimental paradigm with the collective dynamics of our model system and highlight reasons for the ambivalence about the emergence of this phenomenon [11], [64]–[66].

**Figure 6:**
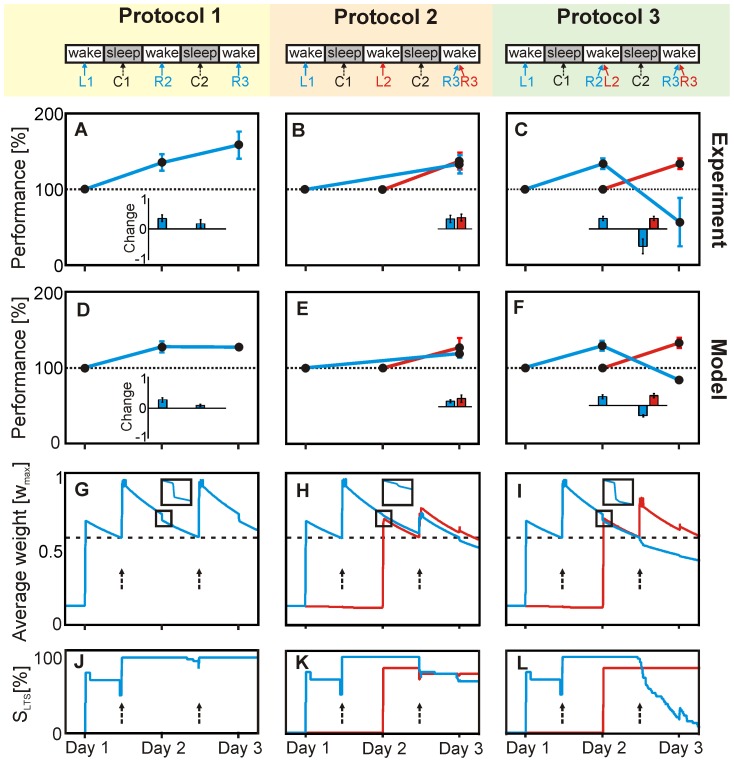
Learning new memory can induce disruption of previously learnt memory in experiment and model. (**A–C**) Experimental results recompiled from Walker et al. [36] showing different memory characteristics in human subjects for a motor learning task over three days. Bar plots (insets) show the relative performance change compared to the previous measured data point. The main panels show the absolute performance change over time. Blue represents the first and red the second memory; ‘L’ is learning, ‘C’ consolidation, and ‘R’ recall of a memory at the respective day. (**D–F**) Performance indices of the model are calculated across *all* neurons of the memory-related sub-population. For details see in Materials and Methods. (**G–I**) Temporal weight development averaged over sub-population. Dashed arrows indicate consolidation at “night”. (**A**) Consolidation leads to significant performance improvement after one night for a single learnt memory (blue). (**B**) Later learning of a second memory (day 2, red) results to improvement of both (blue and red). (**C**) Recall of the first (R2) before learning the second memory (L2) induces strong performance decrease for the first (R3, blue) but not for the second memory (R3, red). (**D–F**) Performance indices (black dots) from the model at the respective points in time follow the same characteristics as human performance in (A–C). (**G,H**) Consolidation leads to recovery of the corresponding weights in both control protocols but (**I**) not for the first (blue) memory when using protocol 3, which leads to a massive reduction of LTS-synapses. (**J–L**) Fraction of memory-related synapses in the LTS-domain.

In a series of elegant experiments, Walker et al. [36] have shown that destabilization of memory happens during a motor learning task. In a control experiment (Figure 6, Protocol 1) human subjects were first trained only on one motor sequence (learning, L1, blue, day one) and then tested once on day two (recall, R2) and day three (recall, R3). Significant improvement in accuracy was observed at day two, but not at day three (Figure 6 A). In the second control experiment (Figure 6, Protocol 2) subjects had been trained on the first sequence on day one (L1, blue) and on a different, second sequence on day two (L2, red), hence 24 h later. Testing was done on day three (R3, blue and red) and performance had improved for both sequences equally (Figure 6 B, blue and red bars). Both observations (panels A and B) were explained [36] by the overnight consolidation (C1, C2) of the memory. In the third experiment (Figure 6, Protocol 3) subjects learnt the first sequence on day one and were – as above – tested on day two (R2, blue) showing the same clear improvement (Figure 6 C, left blue bar). Immediately after testing they had to learn sequence two (L2, red). When re-tested on the third day (R3, blue and red) performance had significantly improved for sequence two but dramatically dropped for sequence one (Figure 6 C, right blue and red bars). This indicates that the second memory interferes with the first but only when the first is activated before the second was learnt.

In our model setting, we performed an identical set of experiments, i.e., with the same learning and testing sequences as used for the human subjects. The model was set up with two cell assemblies, partially overlapping at a corner. Assembly one (blue) was trained on one input sequence and assembly two (red) on another sequence. For recall – as explained above (Figure 2) – we stimulate only a randomly selected subset of 30% of the original neurons. Connectivity and all other parameters were the same as before (Figure 1). Training of either sequence leads to increased synaptic weights which are in the LTS-domain, hence, large enough to allow for consolidation. Consolidation stimuli, C1 and C2, were applied “at night”, where we briefly (three times 15 min) stimulated the whole network (similar to the procedures in Figure 1), as indicated by the dashed arrows in panels G–I. In these panels one can also see the development of the synaptic weights for the first (blue) and the second (red) cell assembly for all three experiments. Performance indices of the model (Figure 6 D–F) are similar to those for the human experiments and we find that data points for the two control experiments match (Figure 6 A,D and B,E). Moreover, also the non-trivial effect on memory disruption is robustly reproduced by the model (Figure 6 C,F). The weight growth normally happening at consolidation C2 is only visible in the control protocols (Figure 6 G,H). By contrast, the readout that happens for protocol 3 at R2 effectively prevents the first memory from consolidation (Figure 6 I).

This phenomenon based on the intrinsic competitive effect arising from activation imbalances already discussed for Figure 2 (see inset in panel C) above. This can be seen in panel G here (see box with magnification), as the recalls R2 and R3 yield a reduction of the average weight curve, without inducing transitions from the LTS- to STS-regime. Learning the second memory acts for the first assembly “like a recall”, due to the partial overlap between assemblies. This is visible in panel H (box). Thus, learning a second memory can reduce the average weights of the first one. In panel H all weights are far above threshold and both assemblies can be consolidated. This is different for the last experiment (panel I). Recall R2 together with learning the other sequence L2 pushes the blue curve down more strongly (see box) than in panels G and H such that it has dropped under the bifurcation threshold when consolidation C2 happens. Close beneath threshold we remember that consolidation acts disruptive (see negative parts of the curve in Figure 5 A), which leads to a further weight decrease at time point C2. Panels J–L show the time courses of the fraction of synapses of each cell assembly that are in the LTS-domain, which corresponds to the above discussed effects. We remark that we have set all parameters in this simulation purposefully so that we can in panel I exactly depict the critical bifurcation point, where at C2 the red weights are just above threshold while the blue ones are just below and the first memory is disrupted. This is meant to emphasize that the transition from the LTS- to the STS-regime, which is a qualitative change, is sensitive to the experimental parameters. This might underly the fact that destabilization, which leads to an actual memory disruption, is not always found in real experiments [65], [66]. While recall and learning of other memories can robustly destabilize a memory, it is the relation of the weight-values relative to the bifurcation threshold, which can give rise to memory disruption (or not). A detailed parameter analysis of the destabilization phenomenon, confirming its robustness, is provided in the supplemental information (Figure S7 in Text S1). This analysis shows that only, if weights are too big or stimulation for recall is too broad and not competitive enough, transitions from the LTS- to the STS-domain do not happen as the system will not travel through the bifurcation. We remark that several recalls briefly after each other affect the same subset of synapses and, therefore, a destabilized memory can not be destabilized further by applying more recalls.

**Figure 7:**
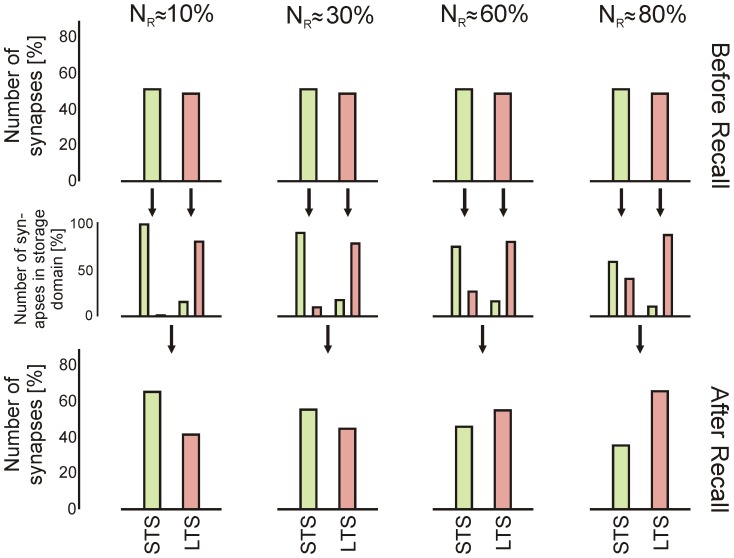
Transitions of synapses between LTS- and STS-regime for different degrees of cell assembly overlap related to the fraction of reactivated neurons during recall. Columns present the fraction or overlap 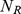 of activated neurons (randomly chosen) in percent of assembly size, rows show how many synapses are in the LTS- or STS-domain (STS: green; LTS: red) before (top) and after (bottom) recall. The middle row shows how many synapses have actually changed their role during the recall. Duration of recall is 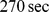.

More specifically, we observe that the overlap between the cell assemblies, related to the fraction of reactivated neurons during recall, is the most critical factor which determines whether one assembly can be destabilized (Figure 7). Zero overlap - trivially so - leads to no disturbance (not shown), small overlap represents the situation which is most strongly susceptible to the disruption of a long term memory (Figure 7, left rows), where more synapses move from the LTS- to the STS-domain than vice versa. By contrast, for a large overlap both assemblies drive each other up into the LTS-domain (Figure 7, right rows). Intuitively this makes sense. Large overlap means that both memories are very similar, hence they might as well begin to couple themselves in an associative (hebbian) way. For small overlap the (dis-)similarity of the memories might rather be “confusing” and an agent (animal/human) might benefit from forgetting one of them not being able to decide whether they are the same or different. It would be interesting to investigate this from a psychophysical point of view. We expect that memory similarity is the crucial factor which determines the capabilities of the system for memory maintenance versus destabilization.

## Discussion

Previous theoretical studies have shown that synaptic scaling could play a key role in neural network dynamics. For instance, synaptic scaling assures competition [67] between synapses at the same dendrite and, therefore, can help to distinguish different inputs [68], [69]. Furthermore, scaling can outbalance neuronal heterogeneities in a way that the performance at working memory tasks is improved [70]. In this study we have shown that synaptic scaling appears a viable candidate mechanism to bridge the large temporal gap between synaptic plasticity (minutes) and synaptic consolidation (days), where we have investigated simulated 24 h sleep-waking cycles. Scaling operates on time scales of hours to days [29] and synaptic plasticity on seconds to minutes [33]. Processes on other time scales, for example short-term plasticity [61], long-term depression (LTD, [34]), or synaptic tagging [22], [23], can influence synapses without great impact on the dynamics of our model, because these mechanisms are “temporally close” to the synaptic plasticity part of the learning rule used here (see Eq. 2). Our analytical and numerical results indicate (Text S1 and [31], [32]) that a different formulation of the synaptic plasticity part will not interfere with the final dynamics as long as the weight-nullcline obeys 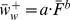 with 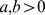 which holds for many generic plasticity rules [31]. This constraint also holds for the more complex dynamics of spike-timing-dependent plasticity (STDP; [35], [71]) as strong neuronal activations lead to long-term potentiation (LTP) independent of the exact timing of spiking [72]–[74]. In an intermediate activity regime we would expect that STDP together with scaling could yield the emergence of even more complex cell assembly structures which could store spatial-temporal patterns [75]–[79]. Over longer time scales (on average) the dynamic of STDP can be simplified by the BCM-rule [47], [80], [81]. This rule consists of an LTP- and an LTD-term and, therefore, the phenomena revealed in this study are maintained (compare also Figure S1 in Text S1). As an important consequence, the bifurcation is preserved under these conditions. Thus, our model with such additional faster synaptic modification mechanisms would exhibit only changed time-courses of the transient synaptic dynamics, for example the learning- or decay times, or more complex structures of cell assemblies, but this would not modify the bifurcation scenario qualitatively and, therefore, the consolidation paradigm presented here. However, not only different plasticity mechanisms can be used, but also the homeostatic term (here, synaptic scaling) could be another (slow) mechanism adapting synaptic weighs. Note, not every homeostatic term (e.g., [46]–[48]) fulfils the above stated weight-constraint.

We considered a class of models of general form (see Materials and Methods). Together with the analytical results this indicates that the phenomenon of synaptic consolidation and differentiation between two storage durations within one network is nearly independent of the underlying network topology (see Figure S2 in Text S1), plasticity rule considered (see above), details of neuronal and network properties, and type of stimuli. The main requirements, which have to be fulfilled, are: (i) a learning rule which guarantees stable synaptic weights depending on the neuronal activity (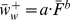) as assured by the combination of LTP and scaling, (ii) leaky, non-linear units (single neurons or ensembles of neurons), (iii) an excitatory recurrent network with, on average, long-range inhibition, and (iv) ‘local’ external stimuli with increased firing rate. Therefore, the bifurcation and consolidation mechanisms described here are not restricted to a certain brain area. Instead, they can occur in every brain area fulfilling the above requirements. Commonly on assumes for memory the neocortex and hippocampus [4], [42], [52], [82]. Furthermore, the area has to have global activations during sleep [6] which could then serve as the consolidation stimulus. Furthermore, the learning stimulus in this model depends on the input frequency. This means that the cell assembly or memory in this model can correspond to a wide variety of long-term memories represented by Hebbian cell assemblies in the brain [14], [15]. This includes declarative as well as non-declarative memory types.

Often (computational) memory models are currently based on attractor neural networks [53], [54], [57], [83]–[85]. In these networks, after the withdrawal of the external input, the activity of a reactivated memory persists for a longer duration [55], [86]. This feature allows for the use of attractor models to reproduce the (relatively) short neuronal dynamics during working memory tasks (up to ten seconds). However, without additional external stimuli these networks are even longer persistently active than the working memory time scale. This means that a reactivated memory in an attractor network will stay active for several minutes or days. Therefore, other mechanisms, as, for instance, inhibitory plasticity [58], are considered to deactivate the recalled memory. All this seems physiologically problematic. By contrast, in our model activity drops back to the background state after a short period (Figure 1) as the memory is not an attractor of the activity dynamics. This is another important property of our system, which combines dynamic behavior with the possibility for synaptic recovery by consolidation. To enable working memory dynamics within this circuit, our model could be extended by the mechanisms of short-term plasticity [61], [87], [88]. However, the drop in activity results in a decay of weights which, due to further mechanisms, could be probabilistic as already proposed by Fusi et al. [89].

The decay of synaptic weights can be avoided by repeatedly delivering brief and global consolidation signals to the network. Here, we assume that such signals can arise during sleep, especially by spindles and ripples [6]. Experimental findings show that, for instance, the disruption of ripples impairs memory consolidation [90] and, furthermore, that synaptic weights are, as in the model, increased after slow-wave sleep or rather spindles [7]. Although we did not include the rich dynamics induced by sleep, our model suggests a potential basis for synaptic consolidation happening during sleep. Furthermore, other experimental studies [25], [26] show that, even six months after learning, memory needs repetitive inductions of plasticity (reconsolidation). The biological mechanisms of this phenomenon are slightly different to initial synaptic consolidation [91]. However, as in this model, the functional properties of these two events are assumed to be similar [13], [63].

The dynamics presented here also yield the fact that the model – similar to the real system – remains susceptible to perturbations and we explicitly reproduced the elusive effect of memory disruption by recall [36]. Similar, drug-induced effects had also been reported in a few studies [11], [37] but others failed to obtain it [65], [66]. Furthermore, learning something new shortly before or after recall seems to increase the chance of perturbing the old memory [12], [13]. This ambivalence is hard to account for with other existing memory models but finds a possible explanation in the bifurcation scenario found here. The bifurcation scenario also predicts that relearning of the disturbed memory should be much faster than before as weights are still larger than without learning. Furthermore, memory similarity (here “assembly overlap”) has a non-trivial effect on consolidation versus destabilization (Figure 7). This is a novel and intriguing prediction which may well be tested in psychophysical experiments.

In general, it seems that memory has to be repeatedly consolidated [4], [25], which could happen during sleep [5], until it is increasingly stabilized. To achieve the latter, systems consolidation, which also begins during sleep [6], performs a transition from a dynamic to a more static memory representation. By this, the stored information is transferred to the neocortex [4]. The process suggested here is capable of repeatedly recovering LTS-candidate synapses, while STS-candidates fade. This may, thus, essentially contribute to providing a stable substrate for systems consolidation and other processes.

## Materials and Methods

### Network

The network consists of a circuit (Figure 1 A) with 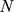 units. Each unit 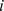 receives an external input 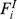 with fixed weight 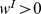. Furthermore, each unit has plastic excitatory connections 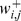 to its 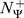 nearest-neighbors 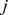 (purple area in Figure 1 A regarding blue unit) and constant inhibitory connections 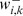 to its 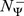 nearest and next-nearest neighbors 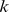 (bluish gray and purple area in Figure 1 A). We remark that the specific layout of this topography is not relevant for the results obtained here (see Figure S2 in Text S1), as long as there is a competition between local excitation and longer-ranging inhibition.

Each neuron 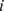 in the circuit is defined by its leaky membrane potential 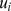 which changes according to
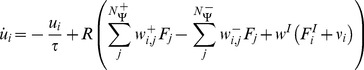
(4)with membrane time constant 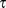, resistance 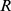, and external input given by 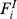 with unchanging input weights 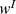. The input is modulated by a noise term 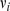 drawn each time step from a normal distribution 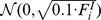 with mean zero and standard deviation 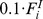. In all simulations the (abstract) membrane potential ranges from values about 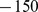 to 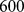.

The membrane potential is non-linearly transformed to a firing rate 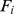 by a sigmoidal-function:
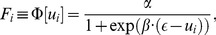
(5)where 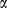 is the maximum firing rate, 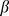 the steepness of the sigmoidal function, and 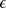 its inflexion point. All parameters combined specify the input-output behavior of the unit.

Only the excitatory synapses 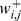 in the second layer are modified using the “Synaptic Plasticity and Synaptic Scaling” (SPaSS)-rule [31]:
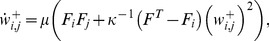
(6)where 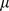 defines the plasticity rate and 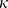 the ratio between plasticity- and scaling rate. The desired ‘target’ firing rate of synaptic scaling is given by 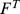. A detailed analysis of the properties of this rule is provided elsewhere [31], [32].

All equations are solved analytically in a mean field approach (see Results section and Text S1) and numerically with the Euler method (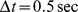). In the following, we provide the parameters used (if not stated otherwise). For numerical simulations, we set 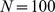, 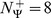, 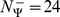, thus, the circuit is a 2-d grid. The inhibitory and projection weights are proportional to the maximal possible weight: 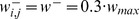 and 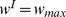 with 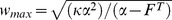. The neuronal parameters are 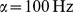, 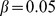, 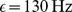, 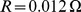, and 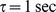. The here shown results are independent of 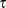. Although, a smaller value would be biological more reasonable, we took 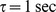 as this avoids numeric instabilities (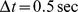). The plasticity parameters are 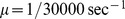, 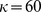, and 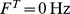. To avoid boundary effects, we used periodic boundary conditions resulting in a toroidal network topology.

### Learning and recall protocol for reconsolidation experiment

In Walker et al. [36] training and recall of memory items differ in the number of blocks each consisting of 30 seconds task followed by 30 seconds rest. Here we use 36 blocks for a training session and 10 blocks for recall. Throughout the task a stimulus of 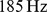 intensity is given to the memory-related neurons (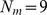). Consolidation signals consist of three blocks with 15 minutes whole network stimulation (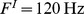) followed by 15 minutes pause. Every time step gaussian noise is added to the external stimuli as mentioned above but with a standard deviation of 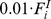.

For Walker et al. [36] as well as for model results all values in the insets of Figure 6 are average values over 10 trials. Data points (black dots) in the main panels have been calculated from the bar plots by us; connecting lines are for graphical reasons only. Performance indices of the model are calculated as time- and space-averages of the synaptic weights across *all* neurons of the respective sub-populations. The time averages have been obtained over five blocks. These are the five last task blocks used for recalls or learning (the learning pulses define the 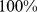 value).

## Supporting Information

Text S1 **Analytical derivations and detailed analyses of cell assembly dynamics.** First, we derive the nullclines of the system and the resulting bifurcation phenomenon. Then, we show that this bifurcation and the related consolidation effect are general mechanisms which hold under different conditions as, for instance, random topology or different synaptic plasticity rule. Furthermore, we provide an analytical derivation of the weight decay without external stimuli and more detailed analyses of memory consolidation and destabilization. At the end of the document is the used Matlab source code for the grid network.
(PDF)
