# Supplementary material for: Synaptic Scaling Enables Dynamically Distinct Short- and Long-Term Memory Formation
Source: PLoS Comput Biol. 2013 Oct 31;9(10):e1003307. doi: 10.1371/journal.pcbi.1003307 (PMC3814677; doi:10.1371/journal.pcbi.1003307)
Supplement: Text S1 — Analytical derivations and detailed analyses of cell assembly dynamics. First, we derive the nullclines of the system and the resulting bifurcation phenomenon. Then, we show that this bifurcation and the related consolidation effect are general mechanisms which hold under different conditions as, for instance, random topology or different synaptic plasticity rule. Furthermore, we provide an analytical derivation of the weight decay without external stimuli and more detailed analyses of memory consolidation and destabilization. At the end of the document is the used Matlab source code for the grid network. (PDF) [file pcbi.1003307.s001.pdf]

Supplemental Information

Derivation of nullclines

Numerical results in Figure 3 A match very well the analytically calculated curves, which we derive here in more detail. First we calculate the weight- and then the activity-nullcline. Finally we determine the fixed points of the system by the intersection of the nullclines.

Weight dynamics

First we are taking Equation 3 and expand it by the sum over all neurons and the excitatory connectivity kernel ($\frac{1}{N}\frac{1}{N_\Psi^+}\sum_i^N\sum_j^{N_\Psi^+}$) at both sides to get the differential equation for the mean field:

$$\dot{w}_{i,j}^+ = \mu\,\left(F_i\,F_j\;+\;\kappa^{-1}\,\left(F^T - F_i\right)\,\left(w_{i,j}^+\right)^2\right)$$

$$\frac{1}{N}\cdot\frac{1}{N_\Psi^+}\sum_i^N\sum_j^{N_\Psi^+}\dot{w}_{i,j}^+ = \mu\,\left(\frac{1}{N}\cdot\frac{1}{N_\Psi^+}\sum_i^N\sum_j^{N_\Psi^+}F_i\,F_j\;+\;\frac{1}{\kappa\,N}\cdot\frac{1}{N_\Psi^+}\;\sum_i^N\sum_j^{N_\Psi^+}\left(F^T - F_i\right)\,\left(w_{i,j}^+\right)^2\right).$$

The fact that inhibition reaches further than excitation allows separation of the network in two (or more) independent ones (memory and control; see main text). Within such a (sub)network the activities of the neurons can be assumed to be equal to the average activity. Thus, $F_i = \bar{F}$:

$$\dot{\bar{w}}^+ = \mu\left(\bar{F}\,\bar{F} + \kappa^{-1}\,\left(F^T - \bar{F}\right)\,\overline{\left(w^+\right)^2}\right).$$

Similarly, we assume that all weights within a (sub)network do not differ much from the average value. Thus, $\overline{\left(w^+\right)^2} \approx \left(\bar{w}^+\right)^2$ and the differential equation turns into

$$\dot{\bar{w}}^+ = \mu\left(\bar{F}\,\bar{F} + \kappa^{-1}\,\left(F^T - \bar{F}\right)\,\left(\bar{w}^+\right)^2\right). \tag{A1}$$

The fixed point ($\dot{\bar{w}}^+ = 0$) of this mean field differential equation yields the weight-nullcline:

$$\bar{w}_w^+ \equiv \bar{w}^+ = \sqrt{\frac{\kappa\,\bar{F}^2}{\bar{F} - F^T}}. \tag{A2}$$

Activity dynamics

Equation 1 expanded by the sum of neurons ($\frac{1}{N}\sum_i^N$) yields:

$$\dot{u}_i = -\frac{u_i}{\tau}\;+\;R\,\left(\sum_j^{N_\Psi^+}w_{i,j}^+\,F_j\;-\;\sum_j^{N_\Psi^-}w_{i,j}^-\,F_j\;+\;w^I\,F_i^I\right)$$

$$\frac{1}{N}\sum_i^N\dot{u}_i = -\frac{1}{\tau\,N}\sum_i^N u_i\;+\;R\,\left(\frac{1}{N}\sum_i^N\sum_j^{N_\Psi^+}w_{i,j}^+\,F_j\;-\;\frac{1}{N}\sum_i^N\sum_j^{N_\Psi^-}w_{i,j}^-\,F_j\;+\;\frac{w^I}{N}\sum_i^N\;F_i^I\right)$$

$$\dot{\bar{u}} = -\frac{\bar{u}}{\tau}\;+\;R\,\left(\sum_j^{N_\Psi^+}w_{(\cdot),j}^+\,F_j\;-\;\sum_j^{N_\Psi^-}w_{(\cdot),j}^-\,F_j\;+\;w^I\,\bar{F}^I\right).$$

As above we assume that the activities within a (sub)network (memory/control) are equal to the average activity. Thus, the differential equation turns into

$$\dot{\bar{u}} = -\frac{\bar{u}}{\tau} + R\,\left(\bar{N}_\Psi^+\,\bar{F}\,\bar{w}^+ \,-\, \bar{N}_\Psi^-\,\bar{F}\,\bar{w}^- \,+\, w^I\,\bar{F}^I\right). \tag{A3}$$

Where we are using $\bar{N}_\Psi^+$ and $\bar{N}_\Psi^-$ as average connectivity values, because in our experiments only parts of the complete network are activated (for example one or two sub-populations of nine neurons). As a consequence, effective connectivity is not homogeneous and neurons at the borders of the active populations have fewer active neurons they connect to. Thus, connection numbers N_Ψ^+ and N_Ψ^- are smaller for border- as compared to core-neurons. Therefore, we need to consider the average number of connections for our calculations as indicated by the small bars ($\bar{N}_\Psi^+$ and $\bar{N}_\Psi^-$).

Setting Equation A3 equals zero yields the following dependency between weight and activity:

$$\bar{w}_F^+ \equiv \bar{w}^+ = \frac{\frac{1}{\tau\,R}\,\bar{u} \,+\, \bar{N}_\Psi^-\,\bar{F}\,\bar{w}^- \,-\, w^I\,\bar{F}^I}{\bar{N}_\Psi^+\,\bar{F}}. \tag{A4}$$

Fixed Points and Bifurcation

The saddle node bifurcation (Figure 3) for the synaptic weights given different input frequencies F^I is obtained by calculating the intersection between the weight- and activity-nullcline ($\bar{w}_w^+ = \bar{w}_F^+$) which provides the fixed points of the system. For this, equation A2 is transposed to $\bar{F}$:

$$\bar{F} = \frac{\bar{w}}{\kappa} \cdot \left(\frac{\bar{w}}{2} \pm \sqrt{\frac{\bar{w}^2}{4} - \kappa\,F^T}\right). \tag{A5}$$

Additionally, $\bar{u}$ can be expressed as a function of $\bar{F}$:

$$\bar{u} = \epsilon - \frac{1}{\beta}\,\log\left(\frac{\alpha - \bar{F}}{\bar{F}}\right). \tag{A6}$$

Both equations have to be inserted in Equation A4 and transposed to a function $\bar{w}(F^I)$. For the resulting equation exists no closed-form, thus, for Figure 3 A we solved it numerically.

Bifurcation and consolidation under different conditions

In the following we will show that the bifurcation and consolidation phenomena (Figure 1 B,C) are guaranteed under different conditions, such as a different synaptic plasticity rule, random topology, and parameter changes.

LTP and LTD

In the main text, we analysed the system for a synaptic plasticity rule consisting of a correlation-term modelling long-term potentiation (LTP). Thereby, we ignored the mechanism of long-term depression (LTD). However, as expected from the analytical calculations, an additional LTD-term does not impair the learning and consolidation dynamics (compare Figure S1 A,B with main text Figure 1 B,C). Here, for the synaptic plasticity part we used the BCM-rule [1] which is a mixture of LTP and LTD: $\dot{w}_{i,j}^{syn.plast.} = F_i\, F_j \cdot (F_i - \Theta)$. We set the parameter Θ, differentiating LTP from LTD, to 10 Hz [2,3]. As the LTP-term in this rule is about F_i-times faster than in the main text, we adapted the time scale μ_{BCM} and the time scale ratio κ_{BCM} by the maximum $F_i^{max} = \alpha$ ($\mu_{BCM} = \mu/\alpha$ and $\kappa_{BCM} = \kappa/\alpha$). All other parameters and inputs are chosen as for Figure 1 B,C in the main text. However, the network dynamics with the BCM-rule are qualitatively the same as with LTP only.

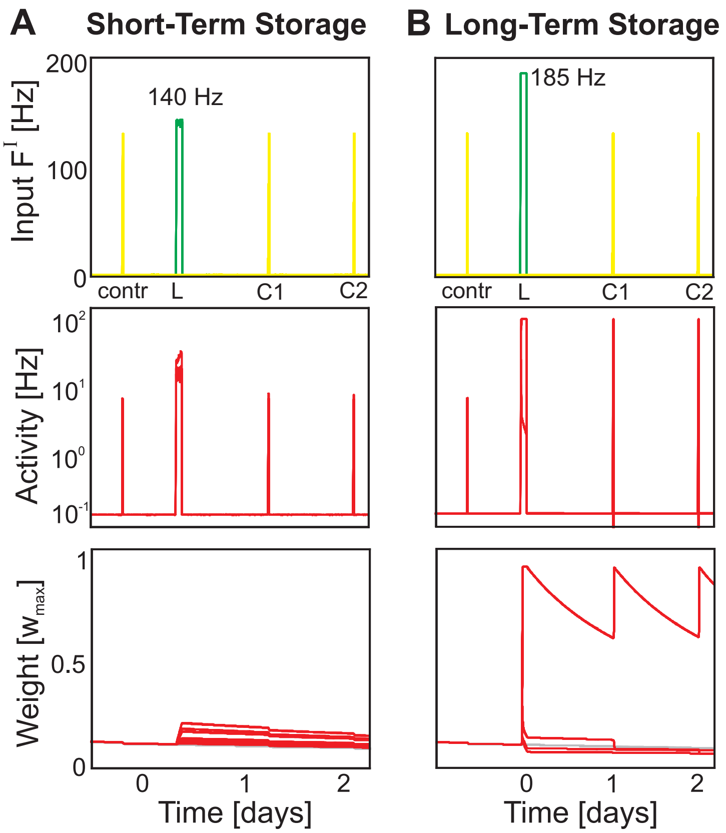

Figure S 1 The dynamic of LTD does not impair the formation of (A) short-term and (B) long-term storage which is consolidated by brief and global stimuli. Here, we used the BCM-rule [1] as combination of LTP and LTD.

Random Topology

The analytical results suggest that only the average number of excitatory and inhibitory connections per unit influence learning and consolidation. Thus, the detailed topology does not influence the dynamics. To show this we used a circuit with randomly seated units on a finite 2-d plane with periodic boundary conditions. Each unit i connects with a certain probability dependent on the distance $d_{i,j}$ to unit j. We used a smaller excitatory probability kernel than the inhibitory kernel:

$$p_{exc/inh} = \exp\left(-\frac{d_{i,j}}{2\,\sigma_{exc/inh}}\right)$$

with $\sigma_{exc} = 0.115$ and $\sigma_{inh} = 0.2$. The parameters are chosen in a way that the average number of connections is comparable to the grid model ($N^+_{grid} = 8$, $N^-_{grid} = 24$): $N^+ = 8.2\pm0.3$ and $N^- = 24.3\pm0.5$ (over 1000 initialisations). Figure S2 A shows, for instance, the topology of one unit (green) to other units (blue: inhibitory connections; red: excitatory connections). A random set of nine neighbouring neurons was externally stimulated (yellow). All parameters and inputs are the same as for Figure 1 B,C. The resulting dynamics of these random networks are qualitatively the same as for the grid network (compare Figure S2 B,C with main text Figure 1 B,C).

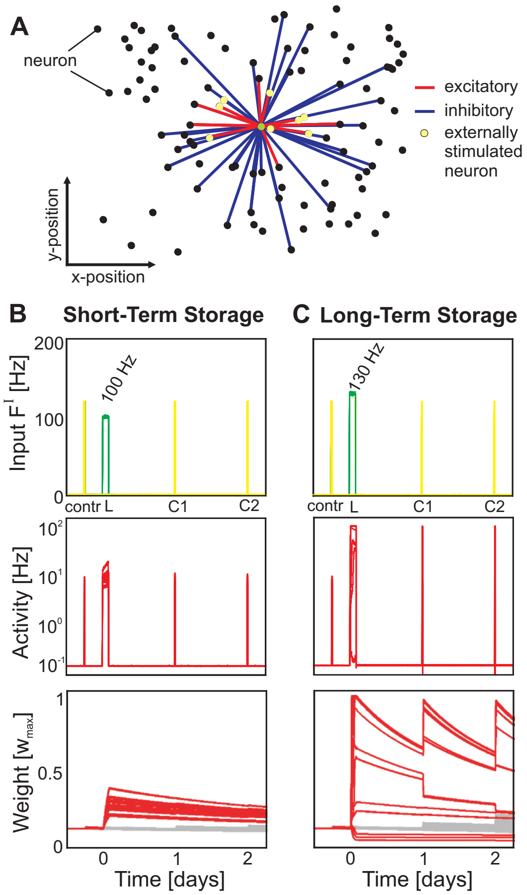

Figure S 2 A random topology (one example unit is shown in A) does not influence the learning and consolidation dynamics (B and C).

Different Parameters

As shown in the main text (Figure 4) a change of parameters does not induce significant differences in the bifurcation diagram. In Figure S3 we show that this also holds true for the dynamics of the circuit. Here, for instance, we changed the desired target value F^T. However, the resulting dynamics are qualitatively the same (compare to Figure 1 B,C in main text).

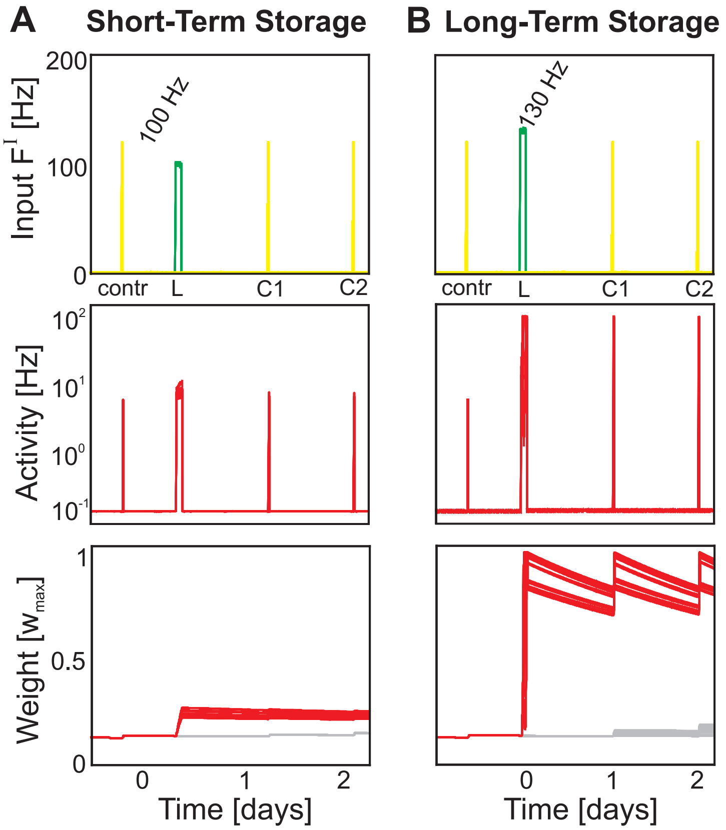

Figure S 3 The usage of different parameters does not impair the dynamics. $F^T = 0.1$, all other parameters and inputs as for Figure 1 B,C.

Passive Weight Decay

Without consolidation all weights will decay (Figure 3 D). This curve of the decay times can be analytically calculated by considering the mean field equation of the weight dynamics (Eq. A1):

$$\dot{\bar{w}}^+ = \mu \left(\bar{F}\,\bar{F} \,+\, \kappa^{-1}\left(F^T - \bar{F}\right)\left(\bar{w}^+\right)^2 \right).$$

During the decay phase the average activity $\bar{F}$ is low, thus, we can assume that the synaptic plasticity part is equal to zero and the mean field equation reduces to

$$\dot{\bar{w}}^+ = \frac{\mu}{\kappa}\left(F^T - \bar{F}\right)\left(\bar{w}^+\right)^2.$$

This differential equation can be solved by separation of variables with initial time point $t_0 = 0$ and weight $\bar{w}^+(t_0) = \bar{w}_0^+$. Thus, the dynamic of the mean weight is

$$\bar{w}^+(t) = \frac{1}{\frac{\mu}{\kappa}\left(\bar{F} - F^T\right)\cdot t + \frac{1}{\bar{w}_0^+}}.$$

As we want to assess the decay times of the weights we transpose this equation to get time t:

$$t = \left(\frac{1}{\bar{w}^+(t)} - \frac{1}{\bar{w}_0^+}\right) / \left(\frac{\mu}{\kappa}\left(\bar{F} - F^T\right)\right).$$

Now, we insert as 'target' weight $\bar{w}^+(t)$ (which has to be reached after time t) the weight value of the controls. Simulations show (Figure 1) that the maximal control weight w^+_{ctrl} is approximately at 0.13, which we, thus, use as target here. Then the time T, which weights need to decay from given initial (learnt) weight w_0^+ to the control weight w^+_{ctrl}, is:

$$T = \left(\frac{1}{w^+_{ctrl}} - \frac{1}{\bar{w}_0^+}\right) / \left(\frac{\mu}{\kappa}\left(\bar{F} - F^T\right)\right). \tag{A7}$$

As can be seen from the bifurcation diagram (Figure 3 A) each synaptic weight value in the STS-regime can be reached by learning. Thus, each initial weight w_0^+ has to be considered here and, therefore, the system has a broad distribution (from seconds to days) of decay times or rather lifetimes of memories (Figure 3 D). This distribution will broaden to infinitely long lifetimes as soon as consolidation signals are given.

Consolidation

Consolidation with different parameters

Figure S4 shows that different durations of consolidation stimuli have little effect on the resulting synaptic weight changes.

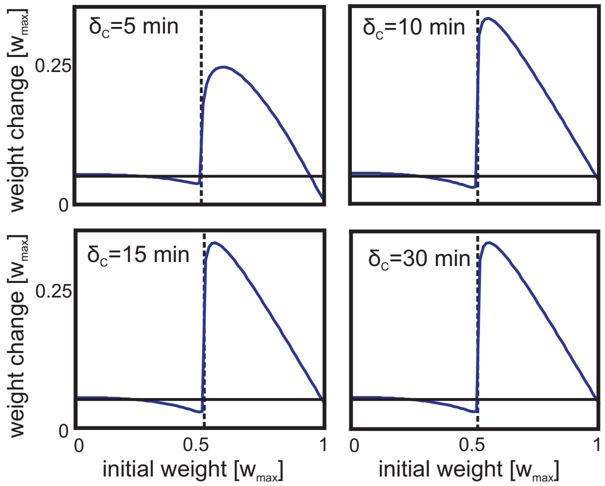

Figure S 4 Longer durations of the consolidation signal above 10 min have all similar effects on the synaptic weight change.

The impact of too late consolidation after learning and previous consolidation

In Figure 5 we show that a consolidation stimuli given too late induce a negative weight change. This is independent of the fact whether the weights had previously been increased by learning or by a consolidation stimulus (Figure S5).

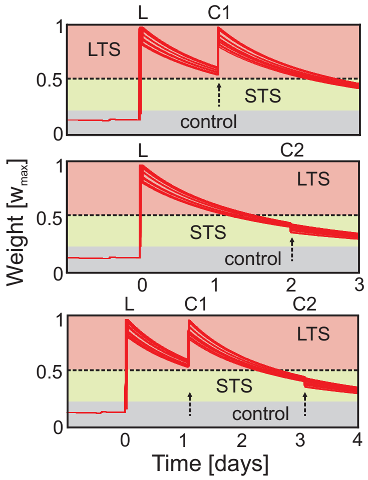

Figure S 5 A too late given consolidation stimulus (arrow) induces weight shrinkage (middle) independent of prior recovery (bottom).

The consolidation cycle

Directly after local learning stimulation (green learning pulses in Figure 1 B, C), LTS- as well as STS-synapses begin to loose strength because only weak background activation is now present until a consolidation signal is delivered (yellow pulses in Figure 1 B, C). The corresponding activity-nullcline for low background activations is plotted in gray in Figure S6 (control). Both types of synapses (STS and LTS) drop from their initially obtained fixed points (Figure 3 B, C) to this nullcline ($*$-markers in Figure S6) and from thereon weights will start to relax back with approximatively $\gamma \bar{w}^2 \bar{F}$ to the crossing with the blue weight-nullcline (fixed point in gray). If time passes without consolidation activation, then, all weights will indeed drop back to the (gray) fixed point nearby zero. Presentation of a strong enough consolidation stimulus changes the picture in a way that the activity-nullcline is shifted from the gray to the yellow one. Therefore, STS- as well as LTS-assemblies will jump up to the yellow nullcline (the activity changes much faster than weights) and follow it upwards with $\mu \bar{F}^2$ (to the new [yellow] fixed point) as long as the stimulus last. Weight decay and recovery (after consolidation stimulus) cyclicly repeat, but one can see that STS-synapses loose more than they gain after every cycle ($\Delta \bar{w}_{STS} < 0$), whereas the LTS-synapses always recover to almost the same value ($\Delta \bar{w}_{LTS} = 0$). This is due to the fact that the recovery depends quadratically on the activity (LTP-term) and LTS-assemblies are clearly more active than STS-assemblies (compare $\bar{F}_{LTS}$ to $\bar{F}_{STS}$).

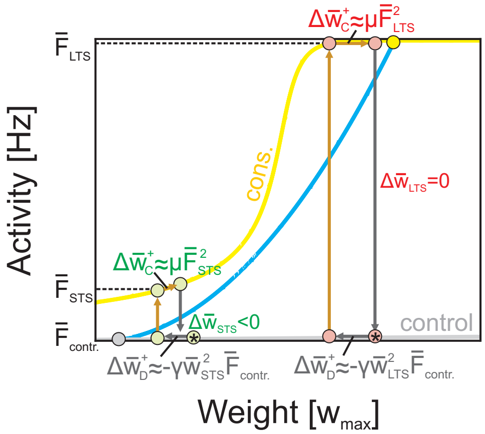

Figure S 6 Schematic of the effect of a consolidation cycle depending on the initial weight values. Blue: weight-nullcline; Gray: activity-nullcline when there is only background input; Yellow: activity-nullcline for the consolidation stimulus. $*$-markers show weight value of STS- and LTS-synapses before consolidation. LTS-synapses recover by consolidation while STS-synapses decay. For more details see supplemental text.

Memory destabilization

Destabilization by recall depends on the activity-dependence of synaptic plasticity and scaling

As mentioned in the main text, the destabilizing phenomenon of a memory recall depends on the imbalance of neuronal activities. For instance, in Figure 2 one neuron in the striped area is externally stimulated and the other not. This (and the neighbor activation) induces two significantly different activities A for the stimulated neuron (yellow in Figure 2 C) and a for the non-stimulated (red; $A > a$). This imbalance results in a small synaptic plasticity term which depends on the correlation (multiplication) of both activities (see below and Eq. 3). Furthermore, the synaptic scaling term for the weakly active neuron is small (incoming synapse), too. However, as A is very large, the synaptic scaling term of the strongly active neuron is significantly larger than the synaptic plasticity term.

$$\begin{aligned}\dot{w}^{+}_{A,a} &= \mu \left(a \cdot A + \kappa^{-1} \left[F^T - A\right] \left[w^{+}_{A,a}\right]^2 \right) \\ &\approx \mu\, \kappa^{-1} \left(F^T - A\right) \left(w^{+}_{A,a}\right)^2 \\ &\approx -\mu\, \kappa^{-1}\, A \left(w^{+}_{A,a}\right)^2\end{aligned}$$

Therefore, we can assume that the weight change is dominated by the negative drive of scaling whereby the synapse shrinks. As this can happen at different positions in the memory-related cell assembly, the memory can be destabilized. Different input intensities yield lower activations and, thus, change the rate of decay but not the effect itself.

Detailed parameter analysis of memory destabilization by partial activation

The recall of a memory item changes the stability of the related cell assembly. Figure S7 provides a details parameter analysis to explain the fact of unbalanced weight transgressions from LTS to STS-domains and vice versa as shown in the main text (Figure 7). We are analyzing the impact of different recall parameters such as stimuli duration and number of reactivated neurons dependent on the initial weight-strength of the cell assembly. Input-target synapses have first been stimulated to grow, reaching different initial values in control-, STS-, and LTS-regime. As the stimulated subset of neurons was chosen randomly, all experiments have been repeated ten times and averaged results have been calculated. In Figure S7 A and C 10% of the neurons have been stimulated. This mimics a recall as well as the activation of another overlapping cell assembly. For panels B and D 30% of the neurons have been stimulated, in panel E 60%, and in panel F 80%. In panels A and B *numerical* weight changes are shown as a function of the initial weight, when applying differently long recalls. Weight growth/shrinkage dominates for small/big weights which may let a synapse change from STS- to LTS-domain or vice versa. Insets show individual curves for 120 sec. recall. Panels C and D show the *fractional* weight changes which gives the relative number of synapses traveling from the LTS-regime to the STS-regime (blue) and vice versa (red). We remark that even in cases with no synaptic transitions the synaptic weights are decreased (panels A and B) which drives them closer to the bifurcation threshold. Recall with 10% overlap leads dominantly to synapses leaving the LTS-domain. For 30% overlap, leaving and entering the LTS-domain is more balanced. If the overlap is larger (60% in panel E and 80% in panel F), recall acts like a learning signal and synapses dominantly move from the STS- to the LTS-domain.

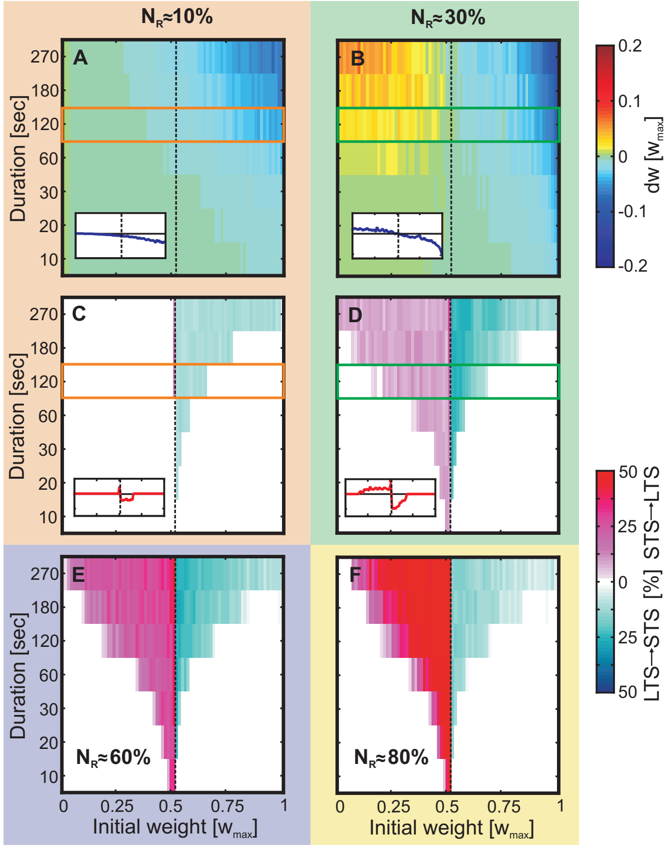

Figure S 7 The effect of partial reactivation of memory-related neurons induces a change of memory stability. The intensity of the recall is 180 Hz. For more details see attended text.

Source Code

In the following we will show the basic source code (Matlab) for Figure 1 B,C:

```
N=100;                              % Number of neurons

shift=10^6;                         % Relaxation Time
TimeSteps=0.7*10^6+shift;           % Total Duration of Simulation
dt=0.5;                             % Step Size

alpha=100;                          % Unit Parameters
beta=0.05;
epsilon=130;
R=0.012;
tau=1;

mu=1/30000;                         % Plasticity Parameters
gma=mu/60;
FT=0;
```

```
inh=sqrt((mu*alpha)/gma)*0.3;          % Synaptic Strength of Inhibitory
                                       % Connections
w_Input=sqrt((mu*alpha)/gma);          % Synaptic Strength of Input
                                       % Projections

InpFrequ=130;                          % Intensity of local Learning Input

NoiseFactor=0.1;                       % Input Noise

Input=zeros(N,SaveTime);               % Vector Initialization
W=(ones(N,N)-0)*10;
U=rand(N,1)*10^-5;
F=U;
counter=0;

%%% Grid Connectivity - Written for N=100
%% Excitatory
C=zeros(N,N);
for i=1:100
    if mod(i,10)~=1
        C(i,i-1)=1;
    end
    if mod(i,10)~=0
        C(i,i+1)=1;
    end
    if i>10
        C(i,i-10)=1;
        if mod(i,10)~=1
            C(i,i-11)=1;
        end
        if mod(i,10)~=0
            C(i,i-9)=1;
        end
    end
    if i<91
        C(i,i+10)=1;
        if mod(i,10)~=0
            C(i,i+11)=1;
        end
        if mod(i,10)~=1
            C(i,i+9)=1;
        end
    end
end


%% Inhibitory
CI=zeros(N,N);
```

```
for i=1:100
        if mod(i,10)~=1
            CI(i,i-1)=-1;
        end
        if mod(i,10)~=0
            CI(i,i+1)=-1;
        end

        if mod(i,10)~=2 && mod(i,10)~=1
            CI(i,i-2)=-1;
            if i>10
                CI(i,i-12)=-1;
            end
            if i<91
                CI(i,i+8)=-1;
            end
        end

        if mod(i,10)~=9 && mod(i,10)~=0
            CI(i,i+2)=-1;
            if i>10
                CI(i,i-8)=-1;
            end
            if i<91
                CI(i,i+12)=-1;
            end
        end


        if i>10
            CI(i,i-10)=-1;
            if mod(i,10)~=1
                CI(i,i-11)=-1;
            end
            if mod(i,10)~=0
                CI(i,i-9)=-1;
            end
        end
        if i<91
            CI(i,i+10)=-1;
            if mod(i,10)~=0
                CI(i,i+11)=-1;
            end
            if mod(i,10)~=1
                CI(i,i+9)=-1;
            end
        end
```

```
        if i>20
            CI(i,i-20)=-1;
            if mod(i,10)~=1
                CI(i,i-21)=-1;
            end
            if mod(i,10)~=1 && mod(i,10)~=2
                CI(i,i-22)=-1;
            end
            if mod(i,10)~=0
                CI(i,i-19)=-1;
            end
            if mod(i,10)~=9 && mod(i,10)~=0
                CI(i,i-18)=-1;
            end
        end
        if i<81
            CI(i,i+20)=-1;
            if mod(i,10)~=0
                CI(i,i+21)=-1;
            end
            if mod(i,10)~=9 && mod(i,10)~=0
                CI(i,i+22)=-1;
            end
            if mod(i,10)~=1
                CI(i,i+19)=-1;
            end
            if mod(i,10)~=1 && mod(i,10)~=2
                CI(i,i+18)=-1;
            end
        end
end

%%% Boundary Conditions - Written for N=100
%% Excitatory
C(1,[100,91,92])=1;
C(10,[99,100,91])=1;
for i=2:9
    C(i,[90-1+i,90+i,90+1+i])=1;
end

C(91,[10,1,2])=1;
C(100,[9,10,1])=1;
for i=2:9
    C(i+90,[-1+i,i,1+i])=1;
end

C(1,[10,20,100])=1;
C(91,[90,100,10])=1;
```

```
for i=1:8
    C(10*i+1,[10*i,10*(i+1),10*(i+2)])=1;
end

C(10,[1,11,91])=1;
C(100,[81,91,1])=1;
for i=2:9
    C(10*i,[10*(i-2)+1,10*(i-1)+1,10*i+1])=1;
end

% Inhibitory
if DNInh==1
    strength=-1;
    CI(1,[10,20,91,92,100])=strength;

    CI(10,[1,11,99,100,91])=strength;

    CI(91,[100,90,1,2,10])=strength;

    CI(100,[81,91,9,10,1])=strength;


    for i=2:9
        CI(i,[90-1+i,90+i,90+1+i])=strength;
    end

    for i=2:9
        CI(i+90,[-1+i,i,1+i])=strength;
    end

    for i=1:8
        CI(10*i+1,[10*i,10*(i+1),10*(i+2)])=strength;
    end

    for i=2:9
        CI(10*i,[10*(i-2)+1,10*(i-1)+1,10*i+1])=strength;
    end
end

if FNInh==1
    CI(1,[89,90,81,82,83,93,99,9,19,29,30])=strength;
    CI(2,[90,81,82,83,84,94,100,10,20,30])=strength;
    for i=3:8    %3-8 and 13-18
        CI(i,80+i-2:80+i+2)=strength;
        CI(i,[90+i-2,90+i+2])=strength;
        CI(i+10,90+i-2:90+i+2)=strength;
    end
    CI(9,[87,88,89,90,81,97,91,1,11,21])=strength;
    CI(10,[88,89,90,81,82,98,92,2,12,22,21])=strength;
```

```
    CI(11,[99,100,91,92,93,9,19,29,39,40])=strength;
    CI(20,[98,99,100,91,92,2,12,22,32,31])=strength;
    for i=2:7   %21-71 and 22-72
        CI(10*i+1,[10*(i-2),10*(i-1),10*i,10*(i+1),10*(i+2)]+9)=strength;
        CI(10*i+1,[10*(i-1),10*(i+3)])=strength;
        CI(10*i+2,[10*(i-1),10*i,10*(i+1),10*(i+2),10*(i+3)])=strength;
    end
    CI(81,[70,69,79,89,99,9,10,1,2,3])=strength;
    CI(91,[80,79,89,99,9,19,20,11,12,13,3])=strength;
    CI(92,[80,90,100,10,20,11,12,13,14,4])=strength;
    for i=3:8   %93-98 and 83-88
        CI(90+i,10+i-2:10+i+2)=strength;
        CI(90+i,[i-2,i+2])=strength;
        CI(80+i,i-2:i+2)=strength;
    end
    CI(99,[7,17,18,19,20,11,1,91,81,71])=strength;
    CI(100,[8,18,19,20,11,12,2,92,82,72,71])=strength;
    CI(90,[61,62,72,82,92,92,1,2,10,8,9])=strength;
    for i=2:7   %30-80 and 29-79
        CI(10*(i+1),[10*(i-2),10*(i-1),10*i,10*(i+1),10*(i+2)]+2)=strength;
        CI(10*(i+1),[10*(i-2)+1,10*(i+2)+1])=strength;
        CI(10*i+9,[10*(i-2),10*(i-1),10*i,10*(i+1),10*(i+2)]+1)=strength;
    end
    CI(12,[100,91,92,93,94,10,20,30,40])=strength;
    CI(19,[97,98,99,100,91,1,11,21,31])=strength;
    CI(82,[70,80,90,100,10,1,2,3,4])=strength;
    CI(89,[61,71,81,91,1,7,8,9,10])=strength;
end

W=W.*C;
WI=CI*inh;


while t~=TimeSteps       % Time-loop
    t=t+1;

        %% Learning Signal to Nine Neurons
        if t>136000+172800+shift && t< 136000+172800+shift+12000; % 2 Hours
                Input([44 45 46 54 55 56 64 65 66],t)=ones(9,1)*...
                InpFrequ+randn(9,1)*NoiseFactor*InpFrequ;
        end

        %% Global, Consolidation Stimulus
        if t==136000+187200+shift+172800 || t==136000+187200+shift+2*172800
                Input(:,t)=ones(N,1)*120;
                counter=1800;      % Global Stimulus for 15 Minutes
        end
        if counter~=0
                Input(:,t)=ones(N,1)*120;
```

```
            counter=counter-1;
        end

        dU=(-U/tau+R*(W*F+w_Input*Input(:,t)))*dt; % Membrane Potential
        dUI=R*(WI*F)*dt;
        U=U+dU+dUI;

        F=alpha./(1+exp(beta*(epsilon-U))); % Non-linear Output Function

        dW=gma*(vT*ones(N,1)-O)*ones(1,N); % Synaptic Scaling
        W=W + (dW.*W.^2)*dt;

        W=W+(mu*F*F')*dt;                     % Synaptic Plasticity
        W=W.*C;
end
```

References

1. Bienenstock EL, Cooper LN, Munro PW (1982) Theory for the development of neuron selectivity: orientation specificity and binocular interaction in visual cortex. J Neurosci 2: 32-48.

2. Dudek SM, Bear MF (1992) Homosynaptic long-term depression in area CA1 of hippocampus and effects of N-methyl-D-aspartate receptor blockade. Proc Natl Acad Sci USA 89: 4363-4367.

3. Graupner M, Brunel N (2012) Calcium-based plasticity model explains sensitivity of synaptic changes to spike pattern, rate, and dendritc location. Proc Natl Acad Sci USA 109(10): 3991-3996.
